# Variations of blood viscosity in acute typhoid fever: A cross-sectional study

**DOI:** 10.25122/jml-2023-0027

**Published:** 2023-10

**Authors:** Salah Al-Windy

**Affiliations:** 1 Department of Biology, College of Science, Baghdad University, Baghdad, Iraq

**Keywords:** typhoid fever, blood viscosity, cardiovascular complications

## Abstract

Typhoid fever (TF) is a systemic infection caused by *Salmonella Typhi (Salmonella Enterica)* transmitted through contaminated water, food, or contact with infected individuals. In various infectious diseases, blood viscosity (BV) is affected by changes in hemoglobin concentrations and acute phase reactants. Inflammatory responses can lead to elevated plasma protein levels and further affect BV. This study aimed to investigate BV changes in patients with acute TF. A cross-sectional study was performed involving 55 patients with acute TF compared to 38 healthy controls. BV and inflammatory parameters were measured in both groups. TF patients showed reduced blood cells compared to healthy controls (p=0.001). Additionally, plasma total protein (TP) levels significantly increased to 10.79±1.05 g/L in TF patients compared to 7.035±1.44 g/L in healthy controls (p=0.03). Hematocrit (HCT) levels were 11.67±2.89% in TF patients and 12.84±2.02% in healthy controls (p=0.07), suggesting a trend towards increased BV in TF patients. Elevated BV is involved in the pathogenesis of different inflammatory and infectious diseases. The increased BV in TF patients may raise the risk of complications. Therefore, monitoring BV might be a crucial tool in TF patients, mainly in the high-risk group, for early detection of cardiovascular complications.

## INTRODUCTION

Typhoid fever (TF) is a systemic infection caused by *Salmonella Typhi (Salmonella Enterica)*, primarily transmitted through contaminated water and food from infected individuals or carriers [1]. Unlike other bacterial infections, TF affects humans exclusively, with no animal reservoirs for the bacteria. After ingesting contaminated food or water, *Salmonella Typhi* replicates initially in the mesenteric lymph nodes and then is transmitted to the blood, spleen, and bone marrow with further proliferation [2].

The clinical symptoms of TF gradually develop, with fever, headache, abdominal pain, vomiting, epistaxis, and bradycardia appearing in the first week. As the infection progresses, fever intensifies, accompanied by the emergence of an abdominal rash in the second week [3]. If left untreated, TF can lead to severe systemic complications, such as intestinal perforation, peritonitis, septicemia, thrombocytopenia, cholecystitis, and encephalitis in the third week [4]. TF diagnosis depends on the clinical features, blood, and bone marrow cultures. However, serological tests like the Widal test may not be reliable due to cross-reactions with other infections [5].

Blood viscosity (BV), a critical factor in blood flow and tissue perfusion, is influenced by various constituents of blood, including hematocrit (HT), rheological properties of red blood cells (RBCs), and plasma protein composition [6]. Elevated HT and plasma protein concentrations can increase BV. Additionally, certain abnormal RBC morphologies, as seen in sickle cell anemia and thalassemia, may reduce BV [7].

Although increased blood viscosity is a possible contributor to the severity of acute typhoid fever, it is not always present during all infections due to other factors, such as chronic infections like hepatitis and cirrhosis [8]. Despite this, it can sometimes be beneficial since it is a natural immune system response against invading bacteria or toxins such as those found in typhoid fever. Increased immune response leads directly to increased inflammation. It produces increased antibodies against invading bacteria or toxins, creating aan army that attacks foreign invaders in the body's tissues and bloodstream. Sometimes, these immune system responses can be beneficial even if they cause temporary damage due to inflammation or local bacterial overgrowth. This happens when a normal immune response occurs at a site where bacteria have invaded via damaged tissue or vessel walls [8, 9].

In various infectious diseases, gamma globulin concentrations and acute phase reactants can also affect BV. Inflammatory changes can elevate plasma protein concentrations, further influencing BV [10]. Elevated BV in severe sepsis can lead to progressive complications and organ injury due to reduced tissue perfusion and vascular damage [11]. Therefore, the primary objective of this study was to investigate alterations in blood viscosity among patients with acute typhoid fever.

## MATERIAL AND METHODS

This cross-sectional study was conducted at the Medical Research Center, Baghdad Medical City, from June 2019 to September 2019 in Baghdad, Iraq. The study involved 55 patients with acute TF aged between 45 and 53 years, as well as 38 healthy controls.

Patients and controls were recruited from private and outpatient clinics. Medical history, physical examination, and routine biochemical and serological tests were conducted for both groups. Informed consent was obtained from all participants. After overnight fasting, 10 mL of blood samples were collected from the patients and stored in a gel tube for analysis.

### Inclusion and exclusion criteria

Patients diagnosed with acute TF were included in the study. Patients with chronic diseases such as hepatic, renal failure, cardiovascular complications, diabetes mellitus, and psychiatric or mental disorders were excluded from this study.

### Measurement of blood viscosity

Blood viscosity was estimated using a specific equation proposed by Duyuler *et al*. The equation depended on the total protein (TP) and hematocrit (HCT). For high shear BV, the equation was BV=(0.12×HCT)+0.17×(TP-2.07), and for low shear BV, it was BV=(1.89×HCT)+3.76×(TP-78.42). HCT was measured as a percentage, TP in g/L, and BV in centipoises (cP). Low shear BV reflects the morphology and aggregation of red blood cells (RBCs), while high shear BV reflects blood flow resistance [12].

### Statistical analysis

The data in this study were presented as mean ± standard deviation (SD). The significance of differences between the two groups was determined using the unpaired Student t-test when the p-value was less than 0.05. To compare more than three variables, analysis of variance (ANOVA) was utilized to assess the significance level [13].

## RESULTS

**Figure 1:**
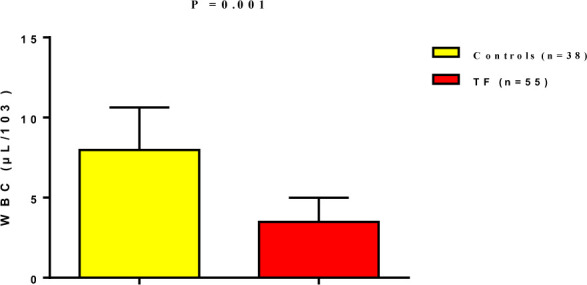
White blood cell levels in patients with typhoid fever compared to controls

In the present study, there were significant differences in various blood parameters between patients with typhoid fever and healthy controls. Specifically, TF patients exhibited a significant reduction in total white blood cell count (3.48±1.51µL/10^3^) compared to healthy controls (7.97±2.66 µL/10^3^) (p=0.001), as shown in Figure 1.

**Figure 2:**
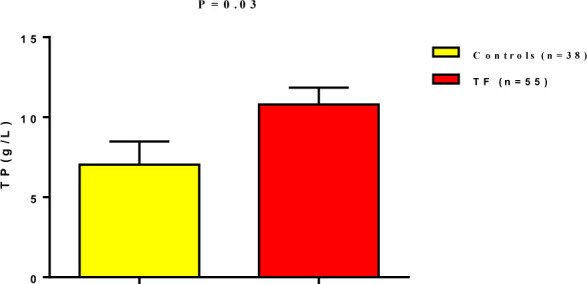
Total protein levels in patients with typhoid fever compared to controls

Furthermore, plasma total protein levels were significantly elevated in TF patients (10.79±1.05 g/L) compared to healthy controls (7.035±1.44 g/L) (p=0.03), as illustrated in Figure 2.

**Figure 3:**
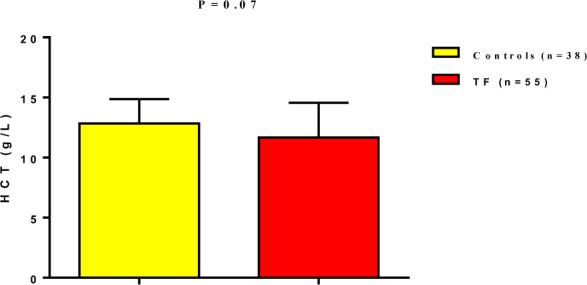
Hematocrit level in patients with typhoid fever compared to the controls

However, hematocrit levels showed no statistically significant difference between TF patients (11.67±2.89%) and healthy controls (12.84±2.02%) (p=0.07) (Figure 3).

**Figure 4:**
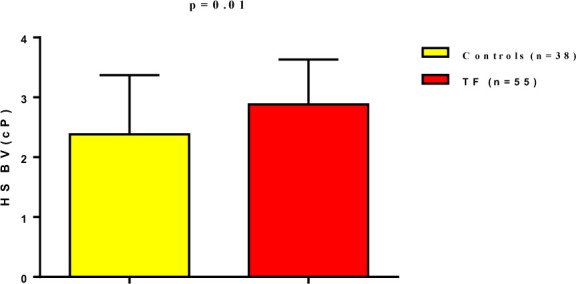
High shear blood viscosity in patients with typhoidfever compared to controls

Therefore, the estimated BV was increased in TF patients compared to the healthy controls. High shear BV was 2.88±0.99 cP in TF patients compared to 2.38±0.75 cP in healthy controls (Figure 4).

**Figure 5:**
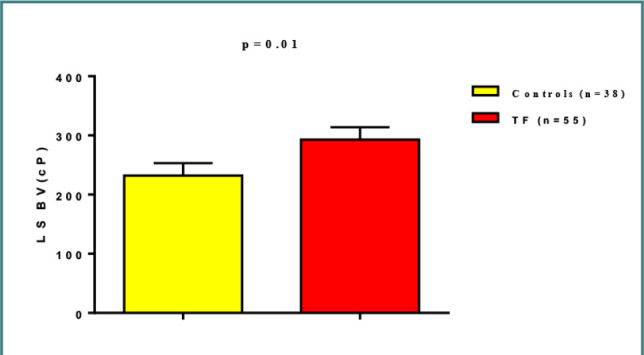
Low shear blood viscosity in patients with typhoid fever compared to caontrols

Low shear BV was 292.89±19.06 cP in TF patients compared to 232.232±21.05 cP in healthy controls (Figure 5).

## DISCUSSION

BV has been implicated in the pathogenesis of various inflammatory and infectious diseases, and its elevation may increase the risk of cardiovascular complications [14]. Our study observed an increase in plasma total protein (TP) levels, which correlated with an elevation in blood viscosity. Hematocrit and plasma viscosity, along with red blood cell (deformability, are considered important determinants of BV) [15]. However, I found that HCT % was not significantly affected in TF patients compared to the controls. Other studies reported that TF might cause the development of anemia. For example, Mohamed *et al*. revealed that iron deficiency anemia might develop in TF patients due to the upregulation of the expression of hepcidin, which impairs iron absorption from the small intestine [16]. Nevertheless, the findings of the present study confirmed that both high and low shears BV were increased in TF patients compared to the healthy controls. BV represents the ratio between shear stress and shear rate that varies with the constant viscosity of shear rates. At low shear, the BV undergoes reversible increment in the veins and arteries due to a change in the erythrocyte deformability [17].

The underlying cause of high BV in TF may involve the development of endothelial dysfunction, high levels of pro-inflammatory cytokines, and increased levels of TP. These changes may induce the development of thrombotic events in TF patients [18]. Coagulation disorders have been reported due to the activation of the coagulation pathway with inhibition of anticoagulant proteins [19]. In turn, thrombotic disorders in TF could be potential causes for increasing BV in severely affected patients. Therefore, monitoring BV might serve as a crucial tool in identifying cardiovascular complications, especially in high-risk TF patients.

The present study had several limitations, including a small sample size, which restraints the generalizability of our findings to larger populations. Additionally, other relevant parameters of BV, such as RBC deformability, plasma viscosity, and acute reactant proteins were not assessed. Nevertheless, this study serves as a preliminary exploration of the relationship between BV and TF.

## CONCLUSION

Elevated blood viscosity has been implicated in the pathogenesis of various inflammatory and infectious diseases, including typhoid fever. In this study, there was a significant increase in BV among TF patients, which suggests a potential association with the development of complications. As BV plays a crucial role in blood flow dynamics, monitoring its levels could be a valuable tool, especially in high-risk TF patients, to identify and manage potential cardiovascular complications.
